# OTTR-seq profiling reveals dynamic tRNA modification landscapes across diverse archaeal species

**DOI:** 10.1186/s13059-026-04158-z

**Published:** 2026-06-18

**Authors:** Jesse S. Leavitt, Henry T. Moore, Thomas J. Santangelo, Todd M. Lowe

**Affiliations:** 1 Department of Biomolecular Engineering, Baskin School of Engineering, University of California Santa Cruz, Santa Cruz, CA 95064 USA; 2 Department of Biochemistry and Molecular Biology, Colorado State University, Fort Collins, CO 80523 USA

**Keywords:** tRNA modification, Archaea, High-throughput RNA modification mapping, Enzyme–substrate coevolution

## Abstract

**Background:**

Transfer RNA (tRNA) modifications are essential for structural integrity, decoding fidelity, and stress adaptation, yet their evolutionary dynamics remain poorly characterized in archaea. Here, we apply Ordered Two-Template Relay sequencing (OTTR-seq), a high-throughput approach that captures full-length tRNAs and modification-sensitive reverse transcription signatures, to systematically profile tRNA modification landscapes across diverse archaeal species.

**Results:**

Across nine archaeal species spanning thermophilic, acidophilic, halophilic, and mesophilic environments, we identify position-specific and clade-dependent patterns of tRNA modifications. We detect coordinated and mutually exclusive methylation at acceptor stem positions 6 and 67 in hyperthermophiles, as well as clade-specific co-modification at positions 10 and 26, which are typically known as tRNA modification anti-determinants. Comparative analyses also reveal lineage-specific divergence in the domain architectures of tRNA methyltransferases, including Trm1, Trm10, Trm11, and Trm14, linking enzymatic evolution to substrate specificity. We further refine known identity elements, such as the G10oU25 pairing, and identify novel structural features that may facilitate or prevent modification.

**Conclusions:**

These findings exemplify the co-evolution of tRNAs and their modifying enzymes, providing new insights into how archaea may fine-tune translation in extreme environments. The broad scope of data and comparative analyses establishes a multispecies framework for future biochemical, mechanistic, and predictive modeling efforts.

**Supplementary Information:**

The online version contains supplementary material available at 10.1186/s13059-026-04158-z.

## Background

Transfer RNAs (tRNAs) play a central role in translating genetic information into functional proteins in all living systems. Post-transcriptional modifications heavily influence tRNA structure and function, modulating the rate of maturation, molecular stability, and the specificity of interactions with aminoacyl-tRNA synthetases, mRNA codons, and a multitude of other cellular components [1–6]. These modifications are differentially conserved at distinct positions within tRNAs, imparting specific properties that vary considerably across the tree of life [7, 8].

Archaea, as the least-studied domain, offers unique insights into how RNA-based regulation and stability evolved, highlighting both the ancient strategies that enabled translation under extreme conditions and the evolutionary roots of RNA modification systems in eukaryotes. Archaea's ecological niches include temperatures approaching 100 °C in deep-sea hydrothermal vents [9], acid hot springs with pH values near 2 [10], and hypersaline environments with salt concentrations exceeding 3 M NaCl [11]. Among methanogens, found exclusively within Archaea, adaptation to anoxic and high-pressure marine environments is evident [12].

For this fascinating branch of life, the complete patterns of tRNA modifications are known for only two species, primarily due to the high cost, difficulty, and low-throughput nature of traditional biochemical characterization of tRNAs. From the comprehensive mapping in *Haloferax volcanii* and *Methanocaldococcus jannaschii* [11, 13]*,* along with partial mapping in *Methanococcus maripaludis*, *Pyrococcus furiosus*, and *Sulfolobus acidocaldarius* [14], it is clear that tRNA modifications contribute to adaptation in diverse extreme environments [15–23]. The chemical stability and structural plasticity conferred by tRNA modifications likely play roles in enabling translation to proceed under these particularly extreme physicochemical conditions.

High-throughput tRNA sequencing has become a powerful tool for quantifying tRNA abundance and expression dynamics, complementing data from traditional mRNA and small RNA-seq approaches that were not designed to measure highly structured and modified RNAs reliably. In addition to profiling tRNA expression, specialized tRNA-seq methods allow the detection of chemical modifications by leveraging the tendency of certain base modifications to disrupt Watson–Crick (W–C) pairing during reverse transcription (RT), which can be captured by quantifying the precise positions of misincorporations or early termination of resulting cDNA strands [24–31]. While mass spectrometry remains the gold standard for the direct chemical identification of modified nucleosides, RT-based sequencing offers a complementary, high-throughput, low-cost, nucleotide-resolution approach ideal for large-scale comparative studies.

One of the newest RT-based RNA-seq methods applied to tRNAs, Ordered Two-Template Relay sequencing (OTTR-seq), enhances detection of modification-sensitive misincorporation events while maintaining full-length tRNA coverage [29, 32]. OTTR-seq utilizes a processive reverse transcriptase with a defined template-switching mechanism, thereby enhancing the uniformity of read coverage across structured RNAs. This makes OTTR-seq particularly well-suited for transcriptome-wide profiling of tRNA modifications, where detecting both conserved and context-dependent modification events requires consistent capture of intact, full-length tRNAs. In archaeal species, where tRNAs are highly structured and often densely modified, this approach enables a broader view of the modification landscape, revealing relationships between modification frequency, tRNA sequence, and enzyme specificity.

Beyond cataloging modifications, understanding the evolution of tRNA-modifying enzymes allows for insights into the rules governing substrate selection. These enzymes can share conserved structural motifs but often exhibit variable domain architectures and phylogenetic distributions, complicating predictions of substrate specificity [33, 34]. Few enzyme-target relationships are governed not only by conserved nucleotide motifs (e.g., G10oU25 pairs) but also by lineage-specific structural features and anti-determinants that influence accessibility or folding of target regions [35, 36].

Much like each aminoacyl-tRNA synthetase (aaRS) recognizes and charges particular subsets of tRNAs based on distinct sequence and structural identity elements [37], tRNA modification enzymes recognize a combination of specific sequence motifs and/or structural elements shared by their target tRNAs. Some modifications, such as N2-methylguanosine (m^2^G) or N2, N2- dimethylguanosine (m^2^_2_G) at tRNA nucleotide position 10 at the base of the D-stem, are essential early in pre-tRNA maturation, mediating the correct folding of the L-shape tertiary structure [38, 39]. Transfer RNA modification pathways are interconnected and exhibit cooperativity with one another, which, in studied cases, influences stress response or leads to disease through modulation of tRNA stability and decoding behavior [3, 5]. To add to this complexity, the precise target specificity of tRNA modification enzymes evolves across diverse phyla, causing uncertainty or inconsistencies between studies on the same family of modification enzymes in different model systems [40].

Among the various tRNA modification types, methylation is the most prevalent, with at least 13 distinct chemical variations of methylated RNA bases shared among all three domains of life [34, 36, 41]. Despite this overlap, modification enzymes vary widely in their protein domain architectures and substrate specificities across the tree of life. While some, like TrmI, function as multi-subunit complexes in eukaryotes but as homotetramers in Archaea [42], others show variability in domain architectures that influence tRNA recognition [43]. These differences impact how modification enzymes selectively recognize and act on diverse tRNA sequences, yet the extent of this variation and its functional consequences remain poorly understood.

Here, we present a survey of archaeal tRNA modifications, revealing novel clade-specific patterns shaped by environmental adaptation and mirrored in enzyme divergence. Using OTTR-seq across nine species, including thermophiles, methanogens, acidophiles, and halophiles, we chart the landscape of RT-detectable tRNA modifications to investigate how modification dynamics evolve across distinct ecological niches. To correlate these new patterns to their enzymes, we examined four AdoMet (SAM)-dependent methyltransferases that target key positions: Trm14 (m^2^G/m^2^_2_G at position 6), Trm11 (m^2^G/m^2^_2_G at position 10), Trm1 (m^2^G/m^2^_2_G/m^2^_2_Gm at position 26), and Trm10 (m^1^A9/m^1^G9). These enzymes span two major classes, with Trm14, Trm11, and Trm1 belonging to class I Rossmann-fold MTases and Trm10 to the class IV SPOUT family [44]. Our integrated analyses highlight position-specific and isoacceptor-dependent variations, coupled with changes corresponding to presence/absence of modification enzyme orthologs or changes to their domain architectures. Together, these data uncover strong co-variation between enzyme families and modifications at key tRNA positions, underscoring the deep co-evolution of archaeal tRNAs with their modifying enzymes, and bootstrapping a probabilistic framework for prediction of modification presence or absence directly from genome sequence.

## Results

### Detection of base-specific misincorporations reveals tRNA modification dynamics across archaea

**Fig. 1:**
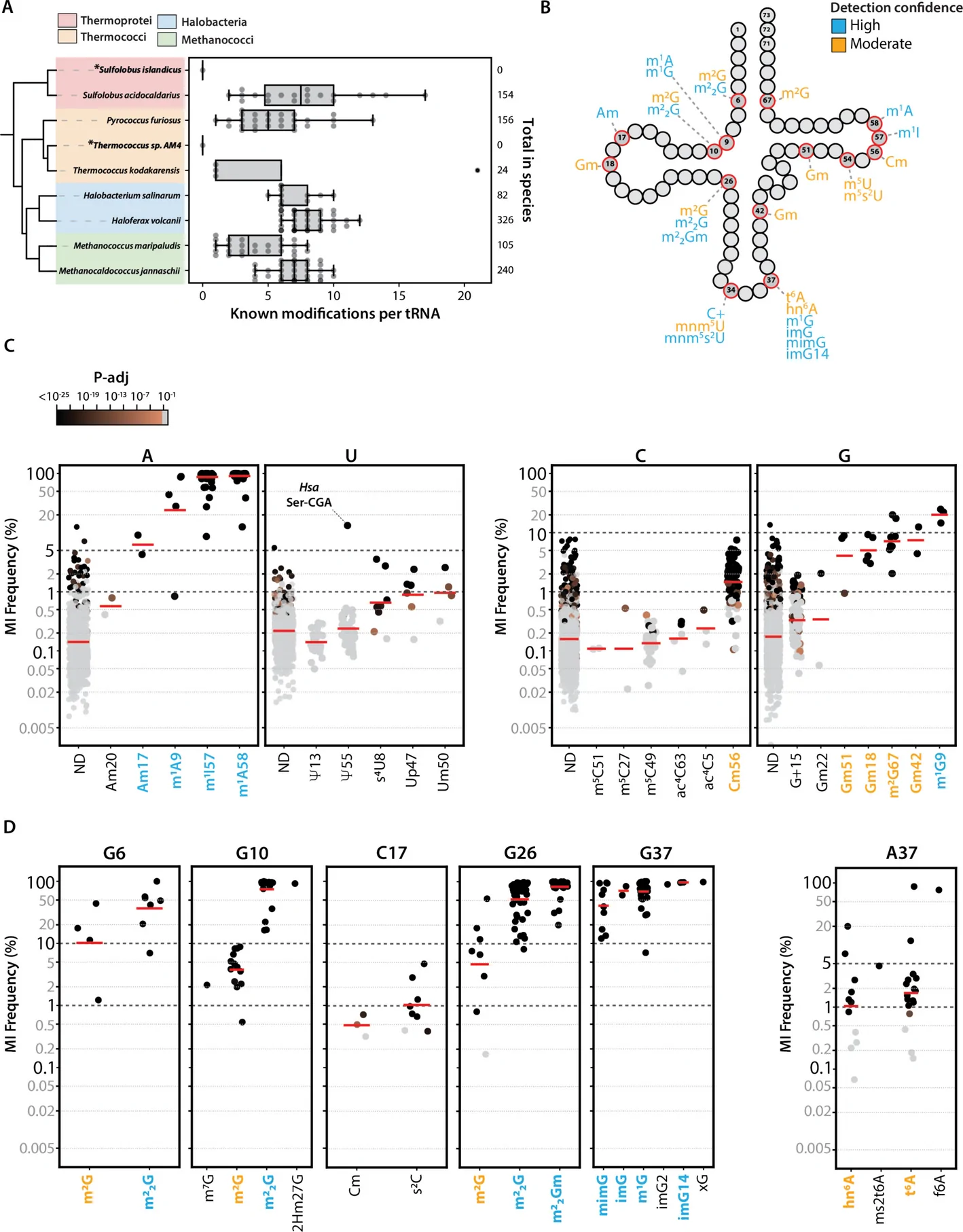
Known archaeal tRNA modifications and their misincorporation (MI) rates. **A** Phylogenetic tree of representative archaeal species, annotated in boxplots, where each datapoint represents a given tRNA with annotated modifications. The number of known tRNA modifications per species was curated from Modomics and literature sources. Species wherein no literature sources define tRNA modifications are marked with an asterisk (*) (**B**) Cloverleaf schematic of tRNA secondary structure indicating detection confidence level at positions of known tRNA modifications across all species. Modifications are color-coded by estimated confidence of detection via OTTR-seq, based on average misincorporation frequency (MI) and adjusted *p*-values. High confidence detection (blue) was classified by base-specific MI rates (mean MI > 10% for cytosine and guanine; mean MI > 5% for adenine and uracil) and an adjusted *p*-value < 0.05. Modifications with significant *p*-values but misincorporation rates below the respective base threshold were classified as moderate-confidence (orange). **C** Log-scale position-specific MI frequencies stratified by nucleotide identity for known modifications and a set of sites classified as ND (Not Determined). ND denotes positions where there is currently no published data supporting a particular modification in the specific species and tRNA context considered (see Methods). Each point represents a position in a specific tRNA, and red lines show mean MI values. Adjusted *p*-values indicate statistical support for misincorporation (copper to black gradient indicates *p*-adj < 0.05; *p*-adj > 0.05 in grey). **D** MI profiles at selected positions where multiple known modification types can occur from the same base (e.g. G6, G10, C17, G26, G37, A37). Each subpanel shows MI distributions stratified by specific chemical modification. Differences in MI frequencies aid in distinguishing closely related modification types such as m^2^G vs. m^2^_2_G at G10. Confidence in detection varies across positions and modification types

We profiled post-transcriptional tRNA modifications across nine archaeal species (Table 1) using OTTR-seq [29] to identify reverse transcription (RT)-induced misincorporation signatures of tRNA modifications (Fig. 1). Known modification sites in seven of these species (*H. volcanii, H. salinarum, M. jannaschii*, *M. maripaludis*, *P. furiosus, S. acidocaldarius,* and *T. kodakarensis*) [11, 13, 14, 45] provided a validated reference for evaluating modification detection (Additional file 1: Tables S1, S2), while additional comparisons with *Thermococcus* sp. AM4 and *Sulfolobus islandicus* enabled assessment of short-term evolutionary conservation (Fig. 1A). We predicted sites of tRNA modification via prominent and statistically significant misincorporation (MI) frequencies, defined as the proportion of sequencing reads at a given nucleotide position that differ from the reference base. While sequencing depth was generally robust across the species, we noted that *S. islandicus* and *M. jannaschii* displayed lower overall tRNA read coverage (Additional file 1: Table S3), a factor considered in predictions (see Methods). Misincorporation frequencies define the extent to which a modification disrupts the incorporation of the cognate nucleotide during reverse transcription, typically by disrupting Watson–Crick (W–C) base pairing, and provide a general proxy for the presence and stoichiometry of modification events. To evaluate detection confidence, we established two key metrics that combine statistical significance and MI frequency thresholds to control both the sensitivity and specificity of modification detection (see Methods). Using this system, the distribution of high- and moderate-confidence modifications is summarized by position (Fig. 1B) and across all tRNAs (Additional file 1: Table S2; Additional file 2: Figs. S1 and Fig. S2).

**Table 1:** Summary of study species and relevant attributes

Species (abbr)	Phylum	Class	Habitat	Temperature/pH preference	Number of tRNA genes
*Sulfolobus islandicus (Sis)*	Crenarchaeota	Thermoprotei	Volcanic hot springs	Thermoacidophile (75 °C, pH 2–3)	46
*Sulfolobus acidocaldarius (Sac)*	Crenarchaeota	Thermoprotei	Terrestrial hot springs	Thermoacidophile (75 °C, pH 2–3)	50
*Pyrococcus furiosus (Pfu)*	Euryarchaeota	Thermococci	Hydrothermal marine sediments	Hyperthermophile (95 °C, pH 6–7)	46
*Thermococcus* sp. AM4 (AM4)	Euryarchaeota	Thermococci	Sea hydrothermal vents	Hyperthermophile (80 °C, pH 6–7)	46
*Thermococcus kodakarensis (Tko)*	Euryarchaeota	Thermococci	Sea hydrothermal vents	Hyperthermophile (85 °C, pH 6–7)	48
*Halobacterium salinarum (Hsa)*	Euryarchaeota	Halobacteria	Hypersaline lakes and salterns	Extreme halophile (40 °C, pH 7)	47
*Haloferax volcanii (Hvo)*	Euryarchaeota	Halobacteria	Hypersaline environments	Moderate halophile (42 °C, pH 7)	52
*Methanococcus maripaludis (Mma)*	Euryarchaeota	Methanococci	Salt marshes	Mesophile (37 °C, pH 7–8)	38
*Methanocaldococcus jannaschii (Mja)*	Euryarchaeota	Methanococci	Marine hydrothermal vent	Hyperthermophile (85 °C, pH 6–7)	37

These MI-detectable tRNA modifications exhibited a range of misincorporation profiles, as categorized by nucleotide (Fig. 1C). We note that relative to adenine and uridine, guanine and cytosine exhibit higher background misincorporation rates for unmodified tRNA positions, which required adjusted MI thresholds for our detection confidence classification (see Methods). Modifications disrupting the W–C face (e.g., m^1^A, m^1^I, m^2^G, m^2^_2_G) consistently showed the highest MI rates, and these deviations involving position-specific and/or isotype-specific patterns revealed biologically relevant differences. For example, while m^1^A58 typically results in near-complete misincorporation, Sulfolobales species showed reduced MI rates at A58 in tRNA^Ala^ (Additional file 2: Fig. S4). Despite lower MI rates, the read identity (patterns of misincorporation) and read depth supported m^1^A modification at this position, suggesting substoichiometric modifications and apparent tRNA-specific regulation (Fig. 1C, Additional file 2: Fig. S3). Similar observations were made for A57 in *P. furiosus* Thr^GGU^_,_ where rates of misincorporation markedly dropped, but the patterns of misincorporation were consistent with m^1^I (Additional file 2: Fig. S4).

Other notable outliers included pseudouridine at position 55, which was consistently undetectable across species, as expected given its minimal impact on reverse transcription. However, in *H. salinarum* Ser^CGA^, we observed an unusually high misincorporation rate and a significant *p*-value for modification at this position. While pseudouridine alone is unlikely to account for this signal, it may reflect the presence of a modified derivative such as m^3^U or m^3^Ψ, which can disrupt the W–C face but has not previously been identified at position 55 (Additional file 2: Fig. S4).

An important observation, consistent with prior tRNA-seq studies [24, 28, 32], was that different modification types occurring at the same position and base had characteristic misincorporation behaviors (Fig. 1D, Additional file 2: Fig. S1). The relative distributions of these rates varied by modification type from the same position. In most cases, this allowed us to make reasonable predictions, choosing between known modifications at the same position (Additional file 1: Table S2). For example, at position 10, the misincorporation frequency for m^2^_2_G was consistently higher than that for m^2^G, allowing for distinction between these modifications. When these patterns are augmented by known positives in previously analyzed species (Fig. 1C), as discussed in the next section, this becomes a powerful method for screening many tRNAs for all modifications that cause misincorporations.

### Homology-based predictions of tRNA modifications identify both conserved and dynamic sites of modification

**Fig. 2:**
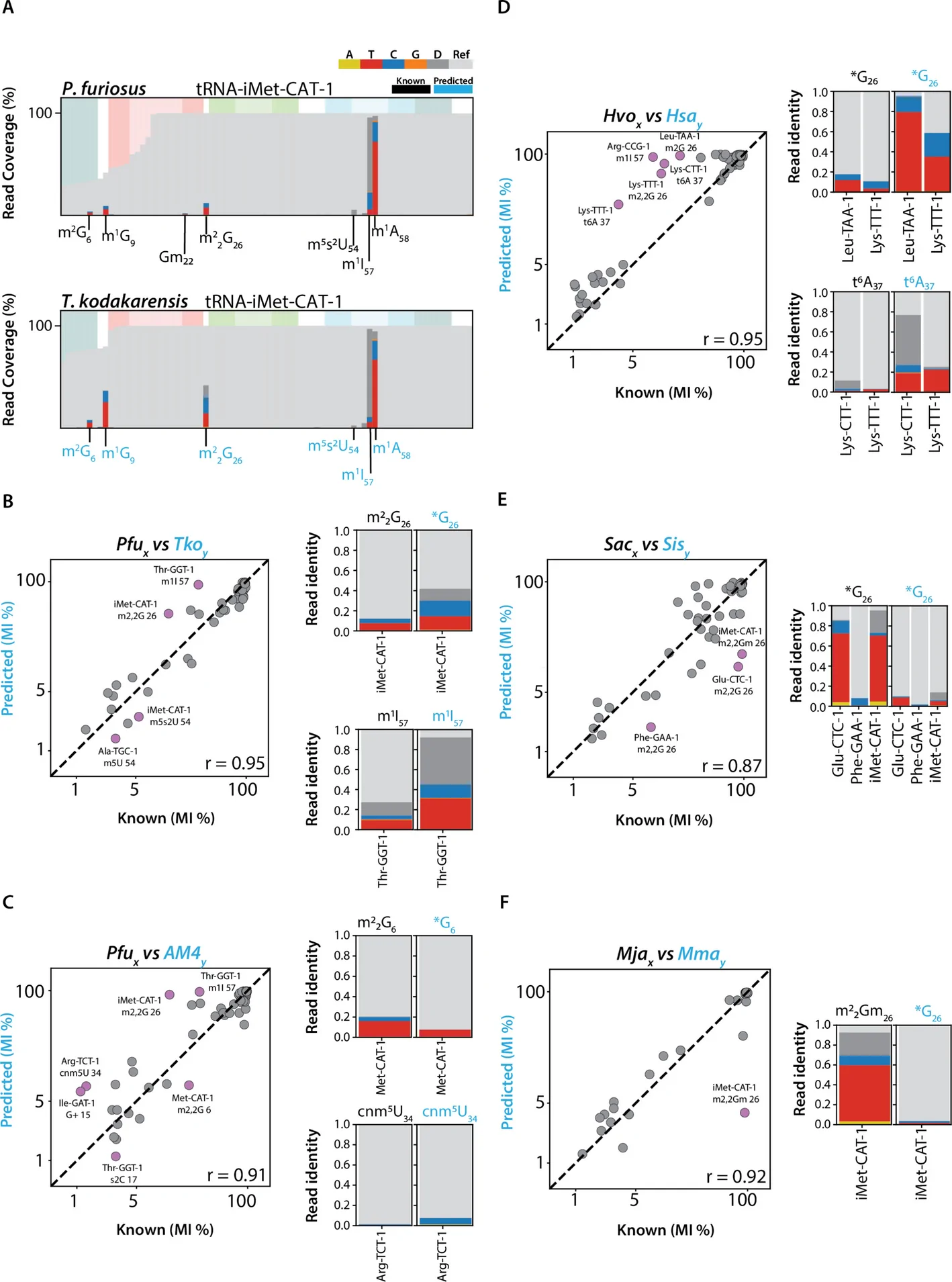
Overview of homology-based tRNA modification predictions. **A** Representative example of homology-based predictions for the initiator methionine (tRNA^iMet^) in *P. furiosus* (top) and *T. kodakarensis (*bottom). The coverage plot depicts the known modification sites indicated (e.g. black) and newly predicted sites highlighted (blue). Mapped-read coverage plot illustrates the read depth and pinpoint sites of modification. **B**-**F** Pearson correlation between known modifications vs homology-predicted modifications. Each point in the scatter plot corresponds to a specific modification type and site from homologous tRNAs. The diagonal line represents perfect correlation (*r* = 1) between known and predicted MI. Outliers (± 2 SD) are highlighted (purple) and annotated with their corresponding tRNA, modification, and position. The read identity (pattern of misincorporation) for select outliers are depicted next to the scatter plot, with known modification position (black) compared to newly predicted (blue) (see all outliers in Supplemental Figures) (**B**) *P. furiosus (Pfu)* vs *T. kodakarensis (Tko)*. Select outliers iMet^CAU^ m^2^_2_G at position 26 and Thr^GGU^ m^1^I at position 57. **C** *P. furiosus (Pfu)* vs *T. species* AM4 *(AM4).* Select outliers Met^CAU^ m^2^_2_G at position 6, and Arg^UCU^ cnm^5^U at position 34. **D** *H. volcanii (Hvo)* vs *H. salinarum (Hsa)*. Select outliers Leu^UAA^ m^2^G, Lys^UUU^ m^2^_2_G at position 26, and Lys^CUU^, Lys^UUU^ t^6^A at position 37. **E** *S. acidocaldarius (Sac) vs S. islandicus (Sis).* All outliers Glu^CUC^ and Phe^GAA^ m^2^_2_G, and iMet^CAU^ m^2^_2_Gm at position 26. **F** *M. jannaschii (Mja)* vs *M. maripaludis (Mma).* Outlier iMet^CAU^ m^2^_2_Gm at position 26. For positions with more than one modification type (e.g., m^2^G/m^2^_2_G_26_) “*” was used. This notation is also used for most predictions, as exact modification types weren’t ascertained

Mapping known modification sites to positions of misincorporations in previously characterized species allowed us to predict their presence in related species lacking experimental annotations (Fig. 2). For example, the known modification profile of tRNA^iMet^ in *P. furiosus* was mapped to concordant sites of misincorporation in *T. kodakarensis*. In this case, we were able to predict all known and detectable modification sites in *T. kodakarensis*, except for 2'-*O*-methylguanine (Gm) at position 22 (Fig. 2A), which has a relatively low misincorporation frequency, even in the reference *P. furiosus* tRNA (Fig. 1C). Additional pairwise comparisons across species further supported the predictive power of misincorporation-based inference of site-specific tRNA modifications. Across comparisons, known and predicted misincorporation frequencies of modified homologous tRNAs were highly correlated (Additional file 1: Table S4; see Methods). For species within the same Class (e.g., Thermococci), Pearson correlation coefficients ranged from 0.87 to 0.95 (Fig. 2B-F, Additional file 2: Fig S5), and this strong agreement indicates that modified homologous tRNAs frequently share conserved misincorporation patterns, reinforcing the value of using known sites of tRNA modification as predictors for tRNA modification patterns in closely related taxa.

However, deviations from expected misincorporation patterns revealed additional layers of biological variation (Fig. 2B-F, purple off-diagonal points). These unexpected profiles were particularly informative, suggesting partial or context-specific modifications in homologous tRNAs between species within the same Class, family, or genus. Notably, for many published modification data sets, there is often no discrimination between fully and partially modified nucleotides, leading to ambiguity when comparing them to our MI frequencies. For example, the known m^2^_2_G in iMet^CAU^ of *P. furiosus* had a lower and more variable pattern of misincorporation compared to the predicted modification of the homologous tRNA in *T. kodakarensis* at position 26 (Fig. 2B; Additional file 2: Fig. S6). Interestingly, the expected modification in *T. kodakarensis* had a larger proportion of RT-induced deletions (Fig. 2B, m^2^_2_G_26_ vs *G_26_). While the triple methylation of guanine (m^2^_2_Gm) is often associated with deletions and higher rates of misincorporation, we broadly define all likely G-derived modifications at position 26 as “*G”, as there are usually two or more modification types that occur at this position, which cannot be consistently distinguished solely through MI frequencies. Similarly, the difference observed at position 57 in Thr^GGU^ between these same two species shows that the patterns of misincorporation are consistent with m^1^I despite variation in rates of misincorporation.

This pattern of divergence was also observed in comparisons with other species. In *P. furiosus* vs *T.* sp. AM4, we identified additional outliers (Fig. 2C, purple points), including the m^2^_2_G in iMet^CAU^ at position 6, a site known to have either m^2^G or m^2^_2_G in other tRNAs. The relatively low misincorporation frequency in *T.* sp. AM4 aligns more closely with m^2^G, prompting us to conservatively annotate this site as m^2^G/m^2^_2_G. Another notable outlier in this comparison was position 34 in Arg^UCU^, where the misincorporation pattern in *T.* sp. AM4 was consistent with cnm^5^U, despite being near the lower limit of detection (~ 1% MI). This highlights the potential to identify U-derived modifications at the wobble position, even when present at low levels, though such predictions warrant cautious interpretation.

Further comparisons between *H. volcanii* and *H. salinarum* (Fig. 2D) revealed similar variations in modification state, which resulted in unresolved prediction ambiguity. In this case the differences in stoichiometry suggest that the “known” reference site may merit re-examination. In *H. volcanii*, the low MI rate and read-identity pattern in Lys^UUU^ are more consistent with m^2^G, matching the known m^2^G in Leu^UAA^. This does not preclude a minority m^2^_2_G fraction in Lys^UUU^ that may explain the reference [11]. By contrast, in *H. salinarum* both Leu^UAA^ and Lys^UUU^ exhibit the elevated MI typical of m^2^_2_G. Because the reference datasets do not report stoichiometry or mixed populations, we conservatively annotate these calls. Additionally, modifications at position 37, such as N^6^-threonylcarbamoyladenosine (t^6^A) in Lys^CUU^ and Lys^UUU^, showed variations in predictions, reflecting either partial modification or differences in species-specific regulation.

A similar trend emerged in *S. acidocaldarius* versus *S. islandicus* analyses (Fig. 2E). Modifications at position 26 in Glu^CUC^, Phe^GAA^, and iMet^CAU^ were all underrepresented in *S. islandicus,* though read identity patterns remained consistent with the expected modification types. These findings suggest that the stoichiometry of modified versus unmodified, rather than the type of modification, may vary between these species. Finally, in *M. jannaschii* vs *M. maripaludis* (Fig. 2F), position 26 in iMet^CAU^ was again identified as an outlier. In this case, the hyperthermophile (*M. jannaschii*) has MI patterns consistent with m^2^_2_Gm, whereas the mesophile (*M. maripaludis*) has patterns consistent with m^2^G.

Together, conserved modifications at positions like 6, 26, and 37 appear to be hotspots for interspecies variation in stoichiometry or type. These comparisons highlight how deviations from expected misincorporation frequencies can predict species-specific differences in modification state. Moreover, read identity patterns support the biological plausibility of these differences, as distinct patterns of misincorporation are often consistent with those of known modifications. Our observations emphasize the caution required in homology-based modification predictions, where careful contextual interpretation is required. For our downstream analyses, we focus on modifications that have moderate to high confidence of detection, and use *N notation at positions where the exact modification type is unable to be inferred.

### Clade-specific patterns reveal coordinated modification in the acceptor stem and core regions

**Fig. 3:**
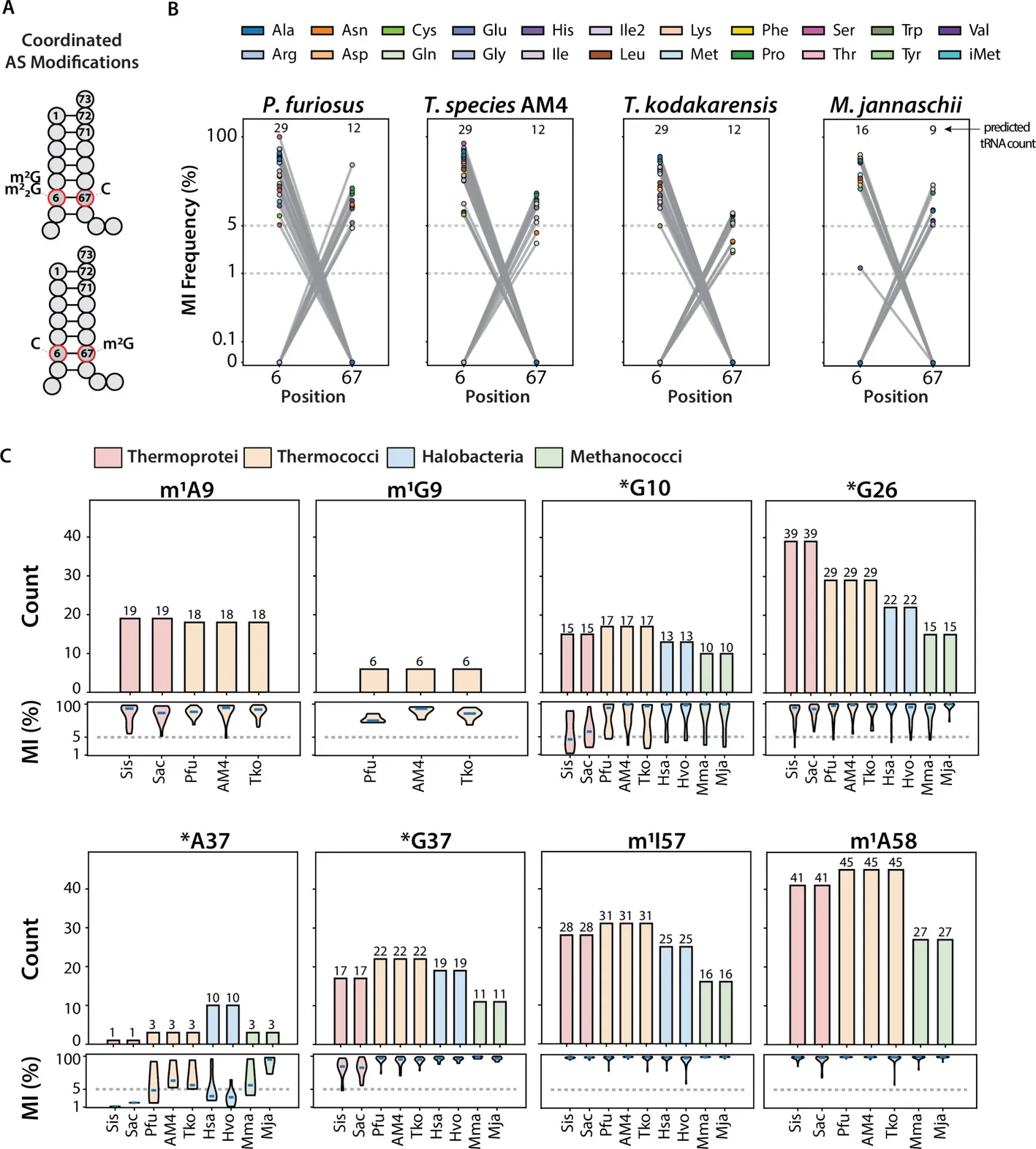
Clade-specific coordination patterns in the acceptor stem and conserved clade-specific tRNA modifications. **A** Schematic representation of coordinated modification patterns at the acceptor stem (AS) of the tRNA secondary structure, highlighting a mutually exclusive pattern between position 6 (typically m^2^G or m^2^_2_G) and position 67 (m^2^G). **B** Paired MI frequencies for position 6 and 67 across four hyperthermophilic archaea: *P. furiosus, T.* sp. AM4*, T. kodakarensis,* and *M. jannaschii.* Each line connects paired MI values for individual tRNAs, with colored dots indicating isotype identity. Only moderate and high-confidence predictions (adjusted *p*-value < 0.05) are shown. Numbers above each panel indicates the total number of predicted modified tRNAs at positions 6 and 67, respectively. A pattern of coordinated modification is consistently observed. **C** Distribution of MI frequencies for select moderate to high confidence tRNA modifications shared across archaeal clades, grouped by phylogenetic class: Thermoprotei (salmon), Thermococci (tan), Halobacteria (blue), and Methanococci (green). Each violin plot summarizes the frequency and variation of a specific modification (e.g. m^1^A9, m^1^G9, m^2^G10, etc.) across species, with counts (above plots) indicating the number of modified tRNAs per group. This highlights the phylogenetic conservation and variability of tRNA modification patterns across lineages

Distinct modification patterns in the acceptor stem and tRNA core regions reveal coordinated signatures that vary by phylogenetic clade. In particular, m^2^G and m^2^_2_G modifications at positions 6 and 67 (base pairing in the acceptor stem) are frequently predicted in hyperthermophilic species (Fig. 3A). While prior studies have reported individual modifications at these positions in select tRNAs from *M. jannaschii* and *P. furiosus* [13, 14], the broader pattern of coordination has not been previously recognized. Our expanded analysis reveals that across multiple species, position 6 is more frequently modified than position 67, with misincorporation rates suggesting a predominance of m^2^_2_G6 over m^2^G6. In contrast, m^2^G67 is less frequent but consistently observed in distinct tRNA subsets (Additional file 1: Table S5; Additional file 2: Fig. S2). The two modifications are not predicted to co-occur within the same tRNA (Fig. 3B), indicating a likely mutually exclusive pattern of modification. This trend is particularly evident in all Thermococci species and *M. jannaschii* (Methanococci). Most G6 conserved tRNAs are predicted to have a modification across these species (Additional file 1: Table S5; Additional file 2: Fig. S2). However, certain tRNAs in Thermococci, such as Glu^UUG^ and Ile^GAU^, possess a C6 (and m^2^G67), precluding the possibility of m^2^G or m^2^_2_G at this position.

Coordination of modification patterns is also observed between positions 10 and 26, two highly conserved sites of modification across archaeal tRNAs. While the predicted modifications conserved in species from Halobacteria and Methanococci tend to occur in mutually exclusive subsets of tRNAs, co-occurrence of modifications at both sites within the same tRNA is found in Thermococci and Sulfolobales (Additional file 1: Table S6; Additional file 2: Fig. S7). This pattern is especially pronounced in Sulfolobales, where multiple tRNAs such as tRNA^Asp^, tRNA^Glu^, and tRNA^Val^ are predicted to carry both m^2^G/m^2^_2_G10 and m^2^_2_G26 (Additional file 1: Table S6; Additional file 2: Figs. S2, S7). In contrast, while Thermococci also exhibits co-modification of position 10 and 26, their modification types differ. With lower rates of misincorporation at position 10, their tRNAs may instead carry m^2^G10 and m^2^_2_G26 (Additional file 2: Fig. S7). These differences are not entirely surprising as these specific clades span two phyla (Crenarchaeota and Euryarchaeota). Their preferences in patterns of modifications suggest evolutionary divergence in enzymatic activity and substrate selection. 

Building on these observations, coordinated modification patterns at 10 and 26 often correspond to specific sequence contexts within tRNAs. Predicted G10 substrates typically base pair with U25, consistent with previous observations [46], while G26 modifications tend to occur in tRNAs lacking this pairing (Additional file 1: Tables S7, S8; Additional file 2 Fig. S8). Interestingly, in Sulfolobales, several tRNAs predicted to be modified at both positions contain G10oU25 but lack the D-arm motifs (e.g., U13oU22, U13-A22) commonly associated with m^2^G10/m^2^_2_G10 in other clades. These findings suggest that the coordination of modifications at structurally proximal positions is reflecting shared structural constraints across archaeal tRNAs, while clade-specific tuning of enzyme–substrate interactions contributes to lineage-specific nucleotide variation.

### Conserved and clade-specific tRNA modifications at functional positions support distinct evolutionary adaptations

Several tRNA positions display broad conservation across archaeal species, although their distribution and frequency vary by lineage and environmental niche (Fig. 3C, Additional file 2: Fig. S2). Modifications at position 10 are widespread across clades, reflecting their proposed functional role in reinforcing the D-stem [46]. Our predictions show the frequency of *G10 (m^2^G or m^2^_2_G) is slightly more prevalent in both Thermococci and Sulfolobales, where over a third of the conserved orthologous tRNAs are modified (Fig. 3C, Additional file 2: Fig. S2). While previous work has shown the importance of m^2^_2_G10 for species growing at elevated temperatures [47], we find that it is also a common modification in mesophilic Halobacteria (28% of tRNAs in *H. volcanii*) and Methanococci (27% of tRNAs in *M. maripaludis*). Interestingly, in Thermococci and Sulfolobales, several tRNAs such as Gly^UCC^ and tRNA^Val^ are predicted to carry *G10 despite lacking canonical sequence features generally associated with this modification, such as the G10oU25 pair (Additional file 1: Table S7).

Position 26 is one of the most consistently modified sites across archaeal species, with nearly all clades displaying high numbers of modified tRNAs (Fig. 3C). Sulfolobales show the highest counts with nearly all tRNAs predicted with *G26 (m^2^G, m^2^_2_G, m^2^_2_Gm), followed by Thermococci, Halobacteria, and Methanococci. While previous work has linked the prevalence of m^2^_2_G26 and its hypermodified derivative m^2^_2_Gm with thermophiles [14, 17], our results show highest modification frequency in acidophiles, indicating that *G26 serves functions beyond thermal adaptation alone. Importantly, not all G26-containing tRNA are modified, particularly in Halobacteria, where iMet^CAU^ and Gly^GCC^ are not predicted to be modified, implying selective targeting by Trm1. In several lineages, predicted *G26 modifications appear mutually exclusive with G10oU25 base pairs, consistent with previously defined anti-determinants [46], though we find that Sulfolobales frequently bypass this rule (e.g., tRNA^Glu^ and tRNA^Phe^; Additional file 2: Fig. S8).

Our results reveal additional patterns that suggest clade-specific evolutionary pressures beyond thermophily alone. For instance, position 9 shows clear phylogenetic divergence (Fig. 3C). Sulfolobales predominantly carry m^1^A9 across nearly all A9-containing tRNAs, whereas Thermococci harbors both m^1^A9 and m^1^G9 (Additional file 2: Fig. S2), reflecting broader substrate recognition by their Trm10 homologs [48]. Notably, neither Halobacteria nor Methanococci exhibit evidence of position 9 modifications, consistent with the absence of detectable Trm10 homologs in these clades.

Modifications at position 37, a universally functional site adjacent to the anticodon, display substantial chemical diversity across archaea. Predicted *x*A37 modifications (typically t^6^A and its derivatives) are largely restricted to Halobacteria, however these are often called with low confidence in our experiments and may not reflect true variations between clades (Additional file 2: Figs. S1 and S2). In contrast, *G37 modifications (m^1^G and wyosine-like derivatives) are confidently predicted and observed to be most prevalent in Thermococci, with nearly half of the conserved orthologous tRNAs being modified. Interestingly, Gly^UCC^ in *P. furiosus* uniquely has a modified G37 (all other species contain A37) and tRNA^Val^ displays frequent G37 and A37 variations from species to species. The distinct distributions of these support clade and species-specific strategies for tuning decoding accuracy and anticodon loop stability [49, 50].

Conserved T-arm modifications at position 57 and 58 are among the most consistently observed modifications across species (Fig. 3C, Additional file 2: Fig. S2). Both m^1^I57 and m^1^A58 are most frequent in Thermococci and Sulfolobales, supporting their proposed role in stabilizing tRNA tertiary structure under extreme conditions [42]. In most lineages, m^1^A58 occurs in all A58 tRNAs, with the exception of Halobacteria which lack this near-universal modification. While m^1^I57 is also widespread, occurring in all A57 tRNAs, its occurrence varies from species to species and even within isoacceptors. For instance, Val^UAC^ in *P. furiosus* has G57, consistent with all tRNA^Val^ among Thermococci, but distinctly has A57 in Val^CAC^ and Val^GAC^ which is modified with m^1^I.

Together, these observations outline a backbone of the archaeal tRNA modification landscape, highlighting core functional sites discernable with our high-throughput method (positions 9, 10, 26, 37, 57, and 58). Patterns of variation may help accommodate structural changes needed due to environmental pressures, ranging from temperature and pH to salinity. The balance between conservation and diversification may help to sustain key modification roles while also allowing enough flexibility for unique and specialized functional roles to undergo selective adaptation. From an RNA modification-based regulation perspective, these sites are of particular interest because they demonstrate key design axes for biologically or synthetically altered tRNA function. Tracing these altered patterns of modification back to the source enzymes becomes a feasible goal with the scale of data gathered in this study.

### Phylogenetic variation in tRNA methyltransferase architectures reveals enzyme specialization across archaea

**Fig. 4:**
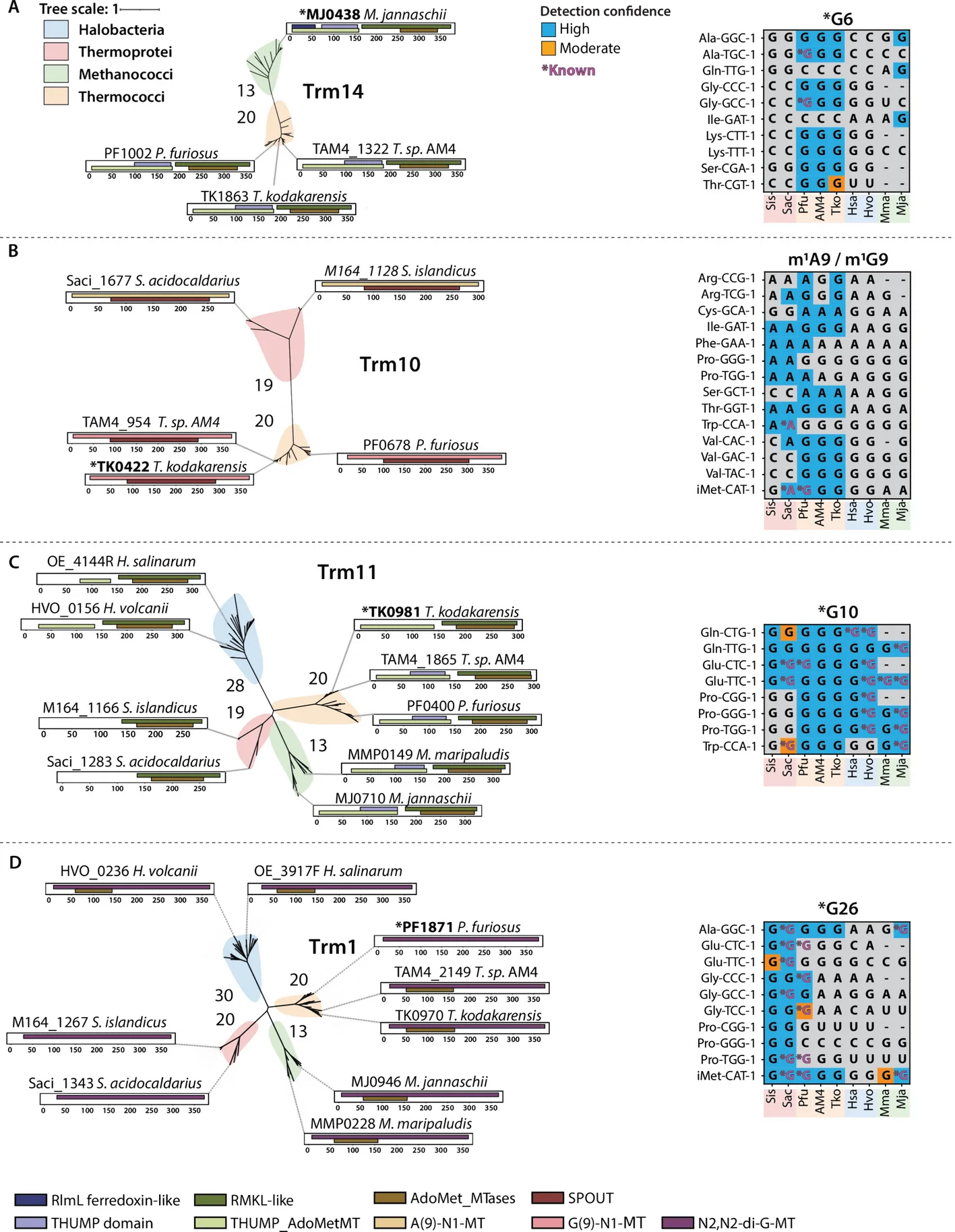
Phylogenetic and structural diversity of predicted tRNA modification enzymes and their target substrates across Archaea. Each panel presents a phylogenetic tree of predicted homologs of a specific tRNA-modifying enzyme (left), alongside a heatmap summarizing the confidence level of predicted substrates across species (right). Trees are constructed from clustered protein sequences colored by archaeal clades: Halobacteria (blue), Thermoprotei (salmon), Methanococci (green), and Thermococci (tan). Domain architecture annotations are shown on the tree branches, indicating key functional domains for three class I MTases (Trm14, Trm11, Trm1) and a class IV SPOUT MTase (Trm10). **A** Trm14 homologs are restricted to Methanococci and Thermococci and are associated with predicted m^2^G6/m^2^_2_G6 modifications in specific tRNA substrates. The corresponding heatmap shows predicted modifications for representative tRNAs across genomes. **B** Trm10 homologs are exclusive to Thermoprotei and Thermococci and are associated with m^1^A9 or m^1^G9 modifications. The heatmap displays inferred substrate preferences for these homologs across tRNAs and illustrates the dual-functional properties unique to Trm10 in Thermococci. **C** Trm11 homologs are broadly conserved, although THUMP domain truncations are observed in Thermoprotei. These homologs are associated with m^2^_2_G10 modifications in select substrates. Divergence is most notable in tRNA^Pro^. **D** Trm1 homologs show widespread conservation, with Thermoprotei variants displaying divergence in the AdoMet domain. Predicted modification patterns at position 26 include m^2^_2_G and other variants such as m^2^G/m^2^_2_Gm (*G26). Positions previously known and annotated as m^2^G reflect sites with adjusted *p*-values > 0.05, indicating lower statistical support. Together, these patterns highlight coordinated evolution of enzyme domain architectures and their target specificity across archaeal lineages

We carried out comparative analysis of tRNA methyltransferase ortholog families to examine enzyme distribution and domain architecture variation alongside clade-specific modification patterns (Fig. 4). We focused on four AdoMet-dependent MTases known to catalyze methylation at specific tRNA positions that showed clade-specific patterns examined above.

Trm14 (m^2^G6/m^2^_2_G6), a THUMP (Thiouridine synthases, RNA methylases and Pseudouridine synthases)-related methyltransferase, occurs exclusively in hyperthermophiles and retains the canonical domains consistent with previously characterized structures [51, 52] (Fig. 4A, Additional file 1: Table S9). Subtle N-terminal differences distinguish clades (e.g., MJ0438 in *M. jannaschii* uniquely has a hit for an RlmL-like domain), however, variations in target substrate are better explained by distinct tRNA features between these species (Fig. 4A). While Trm14 is known to target G6, methylation of G67 remains undetermined. There is evidence of Trm14 involvement in m^2^G67 [45], yet a survey of paralogs identified candidates within these clades that may also be involved (Additional file 1: Table S13).

Contrary to earlier impressions of broad archaeal conservation [48], Trm10 orthologs appear narrowly conserved in Thermococci and Thermoprotei (Fig. 4B, Additional file 1: Table S13). Members of the SPOUT-family are notoriously challenging to model based on primary sequence, however there are detected features compatible with bifunctional G9/A9 N1-methylation in Thermococci, consistent with *T. kodakarensis* TK0442’s dual specificity [53] (Fig. 4B, Additional file 1: Table S10). In Sulfolobales, predicted domain features diverge toward A9-specific residues, and the misincorporation profiles shift accordingly toward A9 targets (Fig. 4B), indicating a phylogenetic split between bifunctional and A-specific Trm10s.

Trm11 (m^2^G10/m^2^_2_G10) is broadly distributed, but Sulfolobales diverge from the canonical domain organization described in *T. kodakarensis* TK0981 [46, 54]. While they retain the C-terminal catalytic domain, they lack the expected N-terminal THUMP tRNA-binding domain (Fig. 4C, Additional file 1: Table S11). This architectural loss coincides with a shift in substrate that co-varies with specific isoacceptors (notably tRNA^Pro^) in our predictions (Fig. 4C). Given there are many known partnerships with Trm112 in *H. volcanii* [55], a modular binding partner (Trm11-Trm112 or analogous factor) may compensate for the missing THUMP in Sulfolobales.

Trm1 (m^2^G26/m^2^_2_G26) lacks a THUMP domain [33, 56] yet, like Trm11, catalyzes mono- then dimethylation. Canonical domain organization is broadly conserved for all predicted orthologs, although profile matches for SAM motifs in the N-terminal region diverge in Sulfolobales (Fig. 4D, Additional file 1: Table S12). Despite subtle differences in architecture, target usage diverges significantly in Sulfolobales, where anti-determinant rules are broken. For instance, both *G10 and *G26 are predicted to co-occur in tRNA^Glu^ (Fig. 4C and D).

Together, our comparison of these key enzyme families establishes a predictive link between domain architecture and target nucleotide choice, further supporting a functional split in Trm10 specificity, and revealing a new THUMP-independent Trm11 with isoacceptor bias. This comparative methodology can be applied to other tRNA modification enzymes extending beyond methyltransferases (e.g., deaminases), as more data is available and methods in high-throughput modification detection continue to improve.

### tRNA sequence and structure features reveal determinants and anti-determinants of modification enzyme targeting

**Fig. 5:**
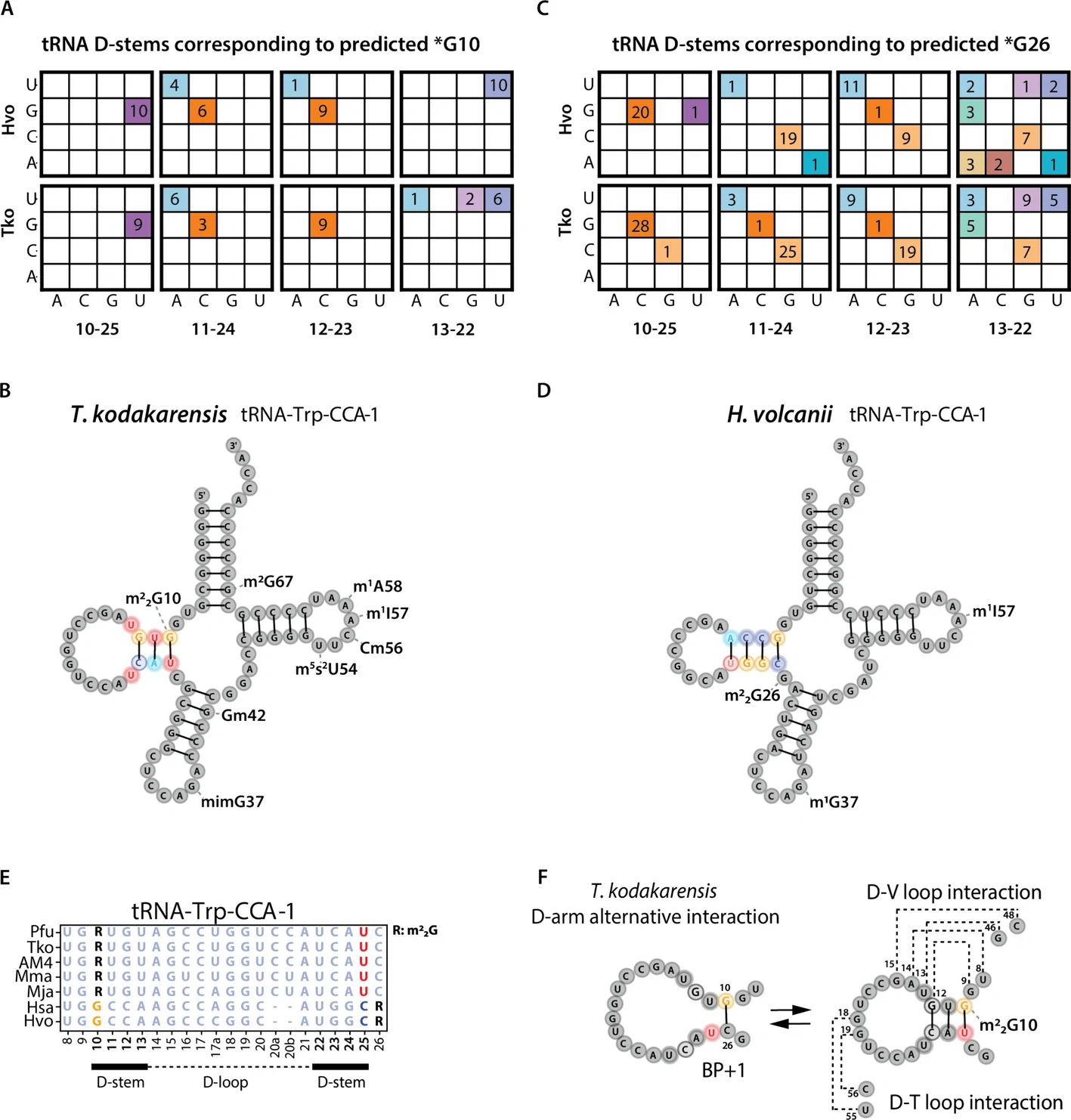
Conserved sequence and structural features correlate with recognition of predicted m^2^_2_G10 and *G26 modified tRNAs. **A** Base-pairing identities in D-stems corresponding to high-confidence predictions of m^2^_2_G10 modifications in *T. kodakarensis* (Tko) and *H. volcanii* (Hvo). Colors represent base-pair combinations and include the number of predicted modified tRNAs for a given pair, such as non-Watson–Crick pairings G10oU25 (purple) and U13oU22 (lavender). **B** Predicted secondary structure of tRNA^Trp^ from *T. kodakarensis*, highlighting key structural recognition features for Trm11-mediated m^2^_2_G10 (G10oU25 and U13oU22). These elements form short, unstable D-arms that facilitate enzyme recognition and modification. **C** Base-pair identities in tRNA D-stems associated with high-confidence predictions of *G26 modifications, where G10 = C25 are conserved and consistent with anti-determinants for m^2^_2_G10. **D** Predicted secondary structure of tRNA^Trp^ from *H. volcanii*, highlighting structural elements and the absence of m^2^_2_G10 with the formation of a stable G10 = C25 W–C pair. **E** Sequence alignment of tRNA^Trp^ D-stem regions species, illustrating conserved structural features, particularly G10oU25 versus G10 = C25. **F** Conceptual illustration of structural instability in the D-arm of tRNA^Trp^ from *T. kodakarensis*. Predicted m^2^_2_G10 modification stabilizes the functional L-shaped tRNA structure by preventing alternative interactions (e.g. a G10 = C26 base pair), promoting proper D-loop and T-loop interactions necessary for canonical folding

Positions with predicted modifications correspond to conserved nucleotide features in specific tRNAs across various species. Likewise, shifts in predicted modifications are linked to changes in nucleotides at distinct positions. Our survey across multiple archaeal clades expands upon previous observations, reinforcing and broadening the understanding of tRNA features critical for modification enzyme recognition. Specifically, the strongest association of these nucleotides aligns with previously characterized recognition features for Trm11, requiring G10oU25 pair in the D-arm [46]. Our broad sampling across archaeal species supports this finding, reinforcing its conserved functional importance. Among Euryarchaeotal species, the frequency of predicted m^2^G10/m^2^_2_G10 modifications correspond with the expected G10oU25 and U13oU22 (Fig. 5A). Moreover, our analysis identifies additional correlations, involving other non-Watson–Crick pairs such as U13oG22 and U13oU22 in Thermococci and Sulfolobales (Additional file 1: Table S7; Additional file 2: Fig. S8).

Non-Watson–Crick pairs, particularly GoU pairs, are known to induce structural variations essential for protein binding without necessitating direct protein-base contact [37, 57]. Our extensive cross-clade analysis further supports that these structural variations may contribute to enzyme recognition. Structurally, residues forming non-Watson–Crick pairs result in shorter, less stable D-stems, which often correspond with the presence of predicted m^2^_2_G10 modifications (Fig. 5B and E). Conversely, we observed absences of these modifications in tRNAs possessing more stable D-stems, further supporting the functional role that this modification has in the structural integrity of tRNAs. For instance, within Halobacteria, the tRNA^Trp^ deviates from the conserved G10oU25 pair, instead forming a G10 = C25 pair, coinciding with the absence of predicted m^2^_2_G10 modification (Fig. 5C, D). This difference highlights the stringent substrate specificity linked to features like U25, essential for facilitating m^2^_2_G10-U25 interactions, but not m^2^_2_G10-C25 interactions.

Similarly, nucleotide features specific to predicted targets of G10oU25 containing tRNAs have been identified as anti-determinants for the action of Trm1, as demonstrated previously in *P. furiosus* [46]. Our results support this relationship among the studied Euryarchaeota species, showing a clear association between the presence of G10oU25 and the absence of m^2^_2_G26 (Fig. 5E). Interestingly, Sulfolobales do not exhibit this exclusion pattern and often show co-modification of m^2^_2_G10 and m^2^_2_G26. Additionally, Sulfolobales show unique targeting by Trm11 of C12 = G23 and A12-U23-containing tRNAs (Additional file 1: Table S7; Additional file 2: Fig. S8). This divergence highlights structural adaptation strategies distinguishing Sulfolobales from other studied archaeal groups. In general, weaker D-arms likely depend on m^2^_2_G10 to stabilize the tRNA in its functional L-shaped conformation [58]. In a specific example of the tRNA^Trp^ of *T. kodakarensis*, we see how m^2^_2_G10 may prevent an alternative interaction between G10 = C26 and likely plays a role in most m^2^_2_G10oU25-containing tRNAs (Fig. 5F). Collectively, this multi-clade analysis significantly extends our current knowledge on interplay between tRNA sequence, structure, and modification enzymes.

## Discussion

Our survey across diverse archaeal species has revealed distinct modification patterns shaped by a combination of environmental pressures and shared ancestry. By interrogating high-throughput RNA sequencing datasets alongside genome-wide protein set analyses, we have expanded our understanding of post-transcriptional tRNA modifications, uncovering novel relationships, hypothesized environmental adaptations, and extensive new data supporting the coevolution of tRNA substrates with their modifying enzymes.

One surprising finding is the coordination of m^2^G/m^2^_2_G at positions 6 and 67 in the acceptor stem of hyperthermophiles, correlating with the presence and conservation of Trm14 orthologs. In contrast, the two Sulfolobales species examined here (merely thermophiles growing at 75 °C), lack both the coordinated modification at these positions and detectable Trm14 homologs. However, the broader distribution of Trm14 homologs present among Thermoprotei species with optimal growth > 80℃ suggests that these acceptor stem modifications may extend beyond Euryarchaeota to include hyperthermophilic Sulfolobales. Previous work noted individual modifications at these positions [13], yet our comparative approach clearly frames them as mutually exclusive. Despite their special occurrence in hyperthermophiles, these modifications may not play a role in thermal stability, which typically involves T-loop/D-loop interactions at the external globular corner of the L-shape [59]. Instead, a prior study [14] suggests that m^2^_2_G6-C67 disrupts the W–C base pair, resulting in a pronounced propeller twist and potentially modulating the aminoacylation efficiency of aaRS tRNA charging. Based on our observations of this coordinated pattern, we hypothesize that steric disruptions between m^2^_2_G6-C67 and C6-m^2^G67 could alter or regulate charging efficiencies of specific tRNAs.

Additionally, we identified coordinated modification patterns involving conserved core positions 10 and 26 across multiple archaeal clades. Our results align with, yet also challenge, established models of substrate specificities for Trm1 and Trm11 orthologs. While tRNAs with G10 typically contain a G10oU25 base pair consistent with previous work [46], the co-occurrence of modifications at both positions 10 and 26 in different species presents a more complex picture. Sulfolobales species, for instance, exhibit m^2^_2_G10 and m^2^_2_G26 within the same tRNA^Glu^, despite being previously proposed as anti-determinants in *P. furiosus*, where structural elements required for dimethylation of one site preclude modification at the other. Broader comparisons allowed us to observe differences in Thermococci, where lower rates of misincorporation at position 10 suggest a predominant pairing of m^2^G10 with m^2^_2_G26 that either co-occur or are coordinated in the same tRNA, such as in Asn^GTT^. This diversity suggests that while fundamental roles of core modifications are conserved, the enzyme specificities have diverged between archaeal lineages. These modification patterns can be explained by the coevolution of Trm1 and Trm11 with their substrates. For instance, we observed the Trm11 orthologs in Sulfolobales uniquely lack the THUMP domain, which is critical for tRNA binding through recognition of the CCA tail [33]. This architectural divergence coincides with the unusual frequency of co-occurring modification patterns as well as variable target substrates. It is plausible that this domain loss is compensated by interactions with accessory proteins, such as Trm112, which is required for Trm11 activity in *H. volcanii* [55]. An enzyme complex could possess altered substrate recognition, enabling the bypass of canonical determinants and would otherwise be unsuitable substrates. The unique targeting of tRNAs with C12 = G23 or A12oU23 pairs in the D-stem of Sulfolobales further illustrates enzyme and substrate coevolution produce lineage-specific outcomes.

The structural elements governing modification in the D-stem are deeply conserved, though specific implementations vary. In yeast, the formation of m^2^_2_G26 requires two consecutive GC pairs in the D-stem, whereas the Trm1 homolog in the bacterium *Aquifex aeolicus* can methylate G26 even when the D-stem contains specific AU pairs, such as A11-U24 or U11-A24 [60, 61]. Likewise, recent work on human TRMT1 shows it catalyzes either m^2^G or m^2^_2_G in a substrate-dependent manner governed by base pairs in the D-stem [62]. This highlights a conserved structural code for enzyme–substrate recognition that has been fine-tuned throughout evolution. As a general rule, these core modifications have been found to be the key to maintaining the tRNA L-shaped architecture and resistance to degradation [32]. Methylations, in particular, enhance thermostability by promoting precise H-bonds (e.g., m^1^A favors Hoogsteen pairs, m^2^G/m^2^_2_G favors GoU pairs), improving base stacking, introducing localized positive charges that interact with the phosphate backbone, and reinforcing tertiary interactions [14, 63]. The presence of m^2^_2_G is especially important, as it can disrupt non-canonical base pairings and prevent alternative, non-functional tRNA conformations. Therefore, the co-occurrence of modifications at positions 10 and 26 in Sulfolobales may reflect a unique evolutionary strategy to ensure tRNA integrity in environments of high acidity and elevated temperatures.

While our analysis revealed clear nucleotide determinants for some modifications, it also highlighted different classes of enzymes that appear to rely on alternative recognition strategies. For instance, sites predicted to carry m^2^G6/m^2^_2_G6, m^1^A9/m^1^G9, and m^2^G67 do not exhibit strong evidence for associations with specific nucleotide residues within the tRNA body. This aligns with previous biochemical studies on enzymes such as Trm10, which suggest that substrate preference may be dictated by the overall stability of the folded tRNA rather than by local sequence motifs [64]. It is plausible that for certain enzymes, recognition depends on global interactions with the L-shaped tertiary structure combined with precise positioning at the catalytic site. This distinction demonstrates the diversity of recognition strategies across modification enzymes and emphasizes that inferring determinants for many modifications requires integrating tertiary structural context.

Distinguishing between these modes of recognition requires accurate and comprehensive modification maps, yet our survey also demonstrates how detection is shaped and limited by the inherent biases of RT-based methods. Modifications that disrupt W–C base pairing, such as m^1^A and m^2^_2_G, consistently yield high misincorporation signals and are therefore robustly detected. In very specific cases, strong misincorporations can cause false “shadow” misincorporations directly adjacent to the real modifications and must be filtered out. Conversely, modifications like 2’-*O*-methylations (Am, Cm, Gm, Um), which alter the ribose sugar rather than the base-pairing face, or other common modifications such as pseudouridine (Ψ) and dihydrouridine (D), all cause minimal disruption to reverse transcription and are notoriously difficult to detect without additional specialized chemical treatments of the RNA before reverse transcription. Thus, the coordinated patterns reported here should be viewed as a subset of the total modification network.

These methodological challenges culminate in the classification of many potential modification sites as “Not Determined” (ND), which represent a frontier for discovery. Future work should prioritize validating ND sites using orthogonal approaches, including mass spectrometry for unambiguous chemical identification and direct RNA sequencing (e.g., nanopores) for detecting RT-invisible modifications such as 2'-O-methylations. Investigating how these newly identified methylations contribute to translational fidelity and stress adaptation in archaea would be a valuable next step.

In summary, this work leverages high-throughput data to map the complex landscape of archaeal tRNA modifications, revealing a dynamic interplay of conserved rules and lineage-specific co-evolution. We present a comprehensive experimental analysis that lays the groundwork for future research by contributing detailed information on tRNA modification profiles, substrate preferences, and evolutionary patterns. These results provide a valuable foundation for guiding targeted biochemical validations, structural studies of enzyme–substrate interactions, and the development of mechanistic models that explain modification specificity and coordination across the domains of life.

## Conclusions

This study provides a comprehensive experimental analysis of archaeal tRNA modifications and the enzymes predicted to install them. By combining high-throughput sequencing with comparative genomic analysis, we uncovered coordinated and clade-specific modification patterns at key tRNA positions and linked these patterns to evolutionary changes in enzyme specificity and domain architecture. Our findings extend the known repertoire of recognition elements and anti-determinants of Trm11 and Trm1 homologs, while also revealing instances in which recognition rules are challenged, particularly within the Sulfolobales. These results emphasize co-evolution between tRNA structure, modifications, and their enzymes in the context of extreme environmental adaptation. Beyond mapping, this work establishes a framework for future biochemical validation, predictive modeling, and functional studies of tRNA modifications.

## Methods

### Culture and RNA isolation

Cultures and total RNA samples for this study were obtained from a combination of in-house growth experiments and collaborations. *H. volcanii,* was cultured in our lab following established protocols (Halohandbook), with three biological replicates that indicated reproducibility for downstream analyses (Additional file 2: Fig. S9). Cell pellets of *M. maripaludis, M. jannaschii, S. islandicus, S. acidocaldarius,* and *H. salinarum* were kindly provided by the laboratories of William B. Whitman, Bishwarup Mukhopadhyay, Rachel Whitaker, Sonja Albers, and David Crowley, respectively [12, 65–71]. *T. kodakarensis* and *T.* sp. AM4 were cultured in Tom Santangelo’s lab [66]. All RNA extractions from externally provided cell pellets were performed by Tom Santangelo’s lab. Total RNA from cell pellets were extracted as described previously [23]. Briefly, cells were treated with BAN reagent (Molecular Research Center, Inc., Cat #BN191). RNA was purified with an equal volume of acid phenol:chloroform:isoamyl alcohol (125:24:1, Thermo Fisher Scientific, Cat #AM9722) and centrifuged to separate the aqueous phase from the organic phase. RNA was then precipitated with 1.5 volumes of isopropanol and 0.1 volume of sodium acetate (Sigma Aldrich, Cat #S7899) at −20C overnight. Finally, the precipitated RNA pellets were washed with 75% ethanol and dissolved in nuclease-free water. Total RNA was used as input for sequencing library construction.

### OTTR-seq library construction

Total RNA was used for generating OTTR-seq libraries, as previously described [29]. Slight adjustments to the protocol were made to improve tRNA capture. Briefly, total T4 polynucleotide kinase-treated RNA was 3’ tailed using mutant *Bombyx mori* R2 retroelement reverse transcriptase (BoMoC RT) in buffer containing only ddATP for 90 min at 30 °C, with the addition of ddGTP for another 30 min at 30 °C. This was then heat-inactivated at 65 °C for 5 min, and unincorporated ddATP/ddGTP were hydrolyzed by incubation in 5 mM MgCl_2_ and 0.5 units of shrimp alkaline phosphatase (rSAP) at 37 °C for 15 min. 5 mM egtazic acid (EGTA) was added and incubated for 100 °C for 5 min to stop the reaction and denature GC-rich tRNAs. Reverse transcription was then performed at 37 °C for 30 min, followed by heat inactivation at 70 °C for 5 min. The remaining RNA and RNA/DNA hybrids were then degraded using 1 unit of RNase A at 50 °C for 10 min. cDNA was purified using the MinElute Reaction CleanUp Kit (Qiagen). To reduce adaptor dimers, cDNA was run on a 9% urea-PAGE gel, and tRNA size range (~ 60–115 bp) was cut out and eluted into gel extraction buffer (300 mM NaCl, 10 mM Tris; pH 8.0, 1 mM EDTA, 0.25% SDS) and concentrated using EtOH precipitation. Size-selected cDNA was then PCR-amplified for 12 cycles using Q5 High-fidelity polymerase (NEB #M0491S) and buffer pack with high GC Enhancer (NEB #B9027S). Amplified libraries were then run on a 6% TBE gel, and tRNA size range (~ 60–115 bp) was extracted to reduce adaptor dimers further. Gel slices were eluted into a gel extraction buffer (300 mM NaCl, 10 mM Tris; pH 8.0, 1 mM EDTA) followed by concentration using EtOH precipitation. Final libraries were pooled and sequenced for 150 single-end reads on an Illumina NextSeq.

### tRNA Analysis of eXpression (tRAX)

Sequencing adaptors were trimmed from raw reads using cutadapt, v1.18 [72], and read counts were generated for annotated RNAs using tRAX [73]. Briefly, trimmed reads were mapped to their respective reference databases: *Methanococcus maripaludis* S2, *Methanocaldococcus jannaschii* DSM 2661, *Sulfolobus islandicus* M.16.4, *Sulfolobus acidocaldarius* DSM 639, *Thermococcus kodakarensis* KOD1, *Thermococcus* sp. AM4, *Pyrococcus furiosus* DSM 3638, *Halobacterium salinarum* R1, and *Haloferax volcanii* DS2. Each reference tRAX database includes both the genome sequences and the mature tRNA annotations obtained from Data Release 22 of the Genomic tRNA Database (GtRNAdb) [74]. Because reads map preferentially to the genome if they are pre-tRNAs with flanking sequence and/or introns, pre-tRNA reads were easily identified and separated from mature tRNA reads as part of the tRAX pipeline. Trimmed reads were mapped using Bowtie2 [75], in very-sensitive mode with the following parameters to allow for a maximum of 100 alignments per read to maximize sensitivity for detecting mis-incorporations that represent RNA modifications: –very-sensitive –ignore-quals –np 5 -k 100. However, to minimize false-positives, mapped reads were then filtered to retain only the “best mapping” alignments as tagged by Bowtie2. Raw read counts of tRNAs and other small RNA types were computed using tRNA annotations from GtRNAdb, and annotations from NCBI RefSeq [76]. Raw read counts were then normalized using DESeq2 [77]. Because the archaea in this study have compact tRNA gene sets (numbering 46–47, or fewer; see Table 1 and the GtRNAdb [74]), the likelihood of mis-mapping is zero or extremely low due to these species having a single, unique gene transcript per anticodon, with multiple sequence differences between transcripts. In the few cases where multiple isoacceptor transcripts were highly similar, read alignments were manually inspected for potential mismapping, and we did not find any evidence of such cases.

### tRNA modification analysis

Sites of known tRNA modifications were compiled from Modomics [78] and relevant literature [13, 14, 45, 53]. Modification types for *M. maripaludis*, *M. jannaschii*, *S. acidocaldarius*, *T. kodakarensis, P. furiosus*, and *H. volcanii* were curated in a tab-delimited table (Additional file 1: Table S1) and mapped to their corresponding tRNA sequence alignments. All tRNA nucleotide positions presented in this work refer to Sprinzl positions, a standard numbering system for tRNAs that assigns consistent numbers to conserved positions across all tRNAs [79]. Multiple naming conventions are used to refer to tRNAs which include tDNA locus name and transcript name with superscript anticodon, switching from T → U, however both are equivalent. Position-specific MI frequencies were stratified by nucleotide identity for known modifications and a set of bases classified as ND (Not Determined). ND includes positions where there are currently no published annotations for modification in the specific species studied. This group likely contains a mixture of both modified and unmodified bases, but no prior data exists to support a specific classification. ND serves as a background reference for MI behavior in the absence of modification expectations. Known modification positions were mapped onto orthologous tRNAs in unannotated species within phylogenetic clades, focusing on sites with conserved reference nucleotides and shared rates of misincorporation.

To assess whether observed misincorporation frequencies were statistically significant relative to background, we applied tMAP [32] using aligned tRNA reads from tRAX [73] as input. At each position misincorporation frequency was calculated as the number of non-reference base calls divided by total read coverage of mature tRNAs. A beta distribution was modeled using these empirical counts, incorporating pseudocounts derived from the mean and variance of misincorporation rates at known modified positions. A modification threshold was empirically determined as the 75th percentile of misincorporation rates, stratified by reference base identity. *P*-values were computed using the cumulative distribution function of the beta distribution, representing the probability of observing a misincorporation frequency equal to or greater than the observed value under the null model.

Modification detection relied on two key metrics: the observed misincorporation rate at each position, and the statistical significance (adjusted *p*-values) of these observed rates. We implemented a base-specific thresholding strategy that accounts for background misincorporation rates inherent to each base. Specifically, cytosine and guanine-derived positions tend to exhibit elevated background rates (often exceeding 5%), resulting in statistically significant *p*-values despite the probable absence of true modification (Additional file 1: Table S3). To mitigate false-positive predictions from these elevated baseline rates, we adopted higher confidence thresholds for these bases.

Sites of predicted modification were classified as high-confidence if the MI frequency exceeded 10% for G/C bases or 5% for A/U bases and had statistically significant adjusted *p*-values (< 0.05). Positions with significant *p*-values but MI rates below these base-specific threshold were classified as moderate-confidence sites due to the observed mixture of true and false positives in our training set. This classification strategy maintains sensitivity for modifications capable of inducing reverse transcription (RT) misincorporation while enhancing specificity across variable signal contexts.

We identified a class of recurring false positives, termed “shadow” misincorporations, which appear to arise as artifacts of reverse transcription processivity near upstream modifications or within short repetitive sequences. The clear patterns of these false positives were identified via comparison of our data to previously characterized species’ tRNA modification sets, and all newly detected modifications matching these well-defined patterns were filtered from our predictions.

Variation in modification detection across species, modification types, and positions primarily reflected differences in MI rate distribution within distinct sequence contexts and tRNA positions. Therefore, statistical significance at similar MI rates varied depending on how closely a given rate aligned with its background distribution. Although low read coverage occasionally limited detection sensitivity, the primary determinants of statistical significance were the depth and magnitude of MI at each position.

In instances where multiple modification types are known to occur at the same position, relative misincorporation frequency was used to infer the likely modification. For example, m^2^_2_G generally causes higher misincorporation than m^2^G at position 10, enabling us to discriminate between these two forms in specific archaeal species. This distinction was not observable at position 6 and 26, therefore these positions were only predicted as m^2^G/m^2^_2_G or *G. In some instances, misincorporation patterns were not sufficiently distinct to confidently infer modification type. In such cases, known modification types from closely related species with identical reference bases were used to assign a most-likely modification classification.

### Homolog search and domain analysis

To search for archaeal homologs and compare domain architectures, we collected representative tRNA modification enzymes that were previously characterized [46, 52, 80, 81]. Each representative was queried across 220 archaeal genomes included in GtRNAdb release 22 [74] using BLASTP [82] with default parameters, then results were filtered to select hits with the lowest e-value for each species and representative. Domain analysis and annotation of highest scoring hits was performed using the InterProScan software package (v5.65–97.0) [83] with the FunFam (4.3.0), MobiDBLite (v2.0), NCBIfam (v13.0), PANTHER (v18.0), SuperFamily (v1.75), CDD (v3.20), Pfam (v36.0), SMART (v9.0), PRINTS (v42.0) and PIRSF (v2023_05) databases. As e-values are specific to each InterPro database and each utilizes their own e-value post-processing, Pfam [84] and CDD [85] were used preferably when domains were found. All matches were considered tentative hits.

### Phylogenetic analysis

The evolutionary relationships among each of the tRNA modification enzymes were inferred using maximum likelihood phylogenies derived from clustered BlastP hits of representatives and their subsequent multiple sequence alignments. In brief, hits for each representative were clustered using MMseq2 [86] using the parameters –min-seq-id 0.3 –cov-mode 0. Clustered groups were subset and aligned with MAFFT [87] using default parameters, then concatenated and aligned again using the parameters –retree 1 –maxiterate 0.

All maximum likelihood phylogenetic trees were estimated using IQ-TREE 2 (v2.1.0) [88]. Selection of the best-fit model of amino acid substitution was inferred for the phylogenies using the ModelFinder function in IQ-TREE (LG + F + R6). Custom python scripts were used to prune trees by keeping only BlastP hits of each representative with the lowest e-value in a genome. Trees of each representative and its best-scoring homologs were plotted using the Interactive Tree Of Life [89]. Predicted domain architectures were annotated onto the phylogenies.

### tRNA secondary structure

The R2DT software [90] was used to predict and illustrate tRNA secondary structures. Predicted modifications and tertiary interactions were illustrated on secondary structure images manually.

## Supplementary Information

Additional file 1. “Leavitt et al supplemental data final” showing data for supplementary tables S1-S13.

Additional file 2. “Leavitt et al supplemental figures final” showing supplementary figures S1-S9.

## Data Availability

Sequence data that support the findings of this study have been deposited in Gene Expression Omnibus with accession number GSE295507 [91]. It is now accessible to all.
