# Supplementary material for: OTTR-seq profiling reveals dynamic tRNA modification landscapes across diverse archaeal species
Source: Genome Biol. 2026 Jun 18;27:277. doi: 10.1186/s13059-026-04158-z (PMC13520502; doi:10.1186/s13059-026-04158-z)
Supplement: Supplementary file 1 [file 13059_2026_4158_MOESM2_ESM.docx]

**Fig S1**. Misincorporation frequency (MI) is shown for a range of annotated tRNA modifications grouped by nucleotide and position (e.g., A37, C17, G10, U54). Each subplot displays individual tRNA sites as points, with color indicating the adjusted p-value (color scale at top right). Red bars denote the mean MI for the modification type. Confidence classification: 5% for A/U and 10% for G/C. Sites with MI values above these thresholds and significant adjusted p-values (p-adj < 0.05) are considered high-confidence predictions; those below the threshold but still significant are classified as moderate-confidence. Note that certain modifications, such as m^2^_2_G display consistent distributions of MI, while others show broader variability.

**Fig S2**. Predicted and known tRNA modifications across positions 6, 9, 10, 18, 26, 34, 37, 42, 51, 54, 56, 57, 58, and 67 in archaeal species, with assigned confidence levels. Each heatmap shows nucleotide identities for all annotated tRNA isotypes across archaeal species. Rows represent species (e.g., *M. jannaschii*, *T. kodakarensis*, *H. salinarum*), and columns represent individual tRNA isotypes. Colored boxes denote predicted modifications: high-confidence predictions (blue), moderate-confidence predictions (orange), and known modifications (purple asterisks). Sites without significant misincorporation signals are shown in gray. Positions with ‘-’ indicate a missing tRNA that is not present in a specific tRNA set, or a tRNA that was not captured by sequencing. This comparative map provides a detailed view of how specific modifications are deployed across phylogeny and tRNA substrates, supporting broader conclusions of clade- and identity-specific modification predictions. However, it’s important to note there are limitations in RT-based detection of non-Watson-Crick disrupting modifications, such as the “invisible” m^5^U54, which are not observed despite being “known” in the reference set. A derivative of m^5^U54, that is m^5^s^2^U54, results in low to moderately detectable misincorporations but should still be considered with caution when drawing comparisons and inferring patterns of variation. Similarly, Cm56 does not directly perturb the RT but occasionally has a subtle impact via a sugar pucker of the 3’ carbon on the ribose that is positioned above the plane. This explains the observation made for many Nm modifications and their low to moderately detectable misincorporations.

**Fig S3**. Total tRNA read counts mapped for each tRNA across all archaeal species. Bar plots show the number of reads mapped to each species. Read counts are shown on the y-axis (log scale), and tRNAs are ordered by amino acid and anticodon. These data reflect the sequencing depth and tRNA abundance for each species. Read coverage was robust across most species. Notably, *S. islandicus* and *M. jannaschii* had comparatively low overall tRNA read depth. However, for tRNAs that were captured, misincorporation profiles are independent of coverage.

**Fig S4**. Outliers (marked with *) for Positions 55, 57, and 58, as referenced in Figure 1C. Pseudouridine at position 55 was consistently undetectable across species, as expected given its minimal impact on reverse transcription. However, in *H. salinarum* Ser^CGA^, we observed an unusually high misincorporation rate and a significant p-value at this position. While pseudouridine alone is unlikely to account for this signal, it may reflect the presence of a related or modified derivative, such as m^1^Y, which has been reported at nearby positions, like 54, but may occur at 55 in this specific context. m^1^I at position 57 was consistently detectable, however in *P.furiosus* Thr^GGU^, we observed an unusually low misincorporation rate that suggests only partial modification. G occurs at this position for 7 tRNAs (Arg-TCT, Asn-GTT, etc), showing a lack of MI, for comparison. Similarly, m^1^A at position 58 was consistently detectable; however, for the Thermoprotei *S. acidocaldarius* and *S. islandicus* tRNA^Ala^ , we observed unusually low misincorporation rates, suggesting partial modification or tRNA-specific regulation. There are additional examples of tRNAs with unexpectedly low misincorporation rates such as His-GTG, where the read depth is low which results in reduced rates of misincorporation and lack of expected modification patterns associated with m^1^A58. In addition, for tRNAs with no read read counts the misincorporation is not displayed.

**Fig S5**. Overview of homology-based tRNA modifications predictions. A-E) Pearson correlation between known modifications vs homology-predicted modifications. Each point in the scatter plot corresponds to a specific modification type and site from homologous tRNAs. The diagonal line represents perfect correlation (r = 1) between known and predicted MI. Outliers (± 2 SD) are highlighted (purple) and annotated with their corresponding tRNA, modification, and position. The read identity (pattern of misincorporation) for select outliers is depicted next to the scatter plot, with known modification position (black) compared to newly predicted (blue) (See all outliers in Supplemental Figures). A) *P. furiosus (Pfu)* vs *T. kodakarensis (Tko)*. Select outliers iMet^CAU^ m^2^_2_G at position 26 and Thr^GGU^ m^1^I at position 57. B) *P. furiosus (Pfu)* vs *T. species* AM4 *(*AM4*).* Select outliers Met^CAU^ m^2^_2_G at position 6, and Arg^UCU^ cnm^5^U at position 34. C) *H. volcanii (Hvo)* vs *H. salinarum (Hsa)*. Select outliers Leu^UAA^ m^2^G, Lys^UUU^ m^2^_2_G at position 26, and Lys^CUU^, Lys^UUU^ t^6^A at position 37. D) *S. acidocaldarius (Sac) vs S. islandicus (Sis).* All outliers Glu^CUC^ and Phe^GAA^  m^2^_2_G, and iMet^CAU^  m^2^_2_Gm at position 26. E) *M. jannaschi (Mja)* vs *M. maripaludis (Mma).* Outlier iMet^CAU^ m^2^_2_Gm at position 26. For positions with more than one modification type (e.g., m^2^G/m^2^_2_G_26_), “x” was used. This notation is also used for most predictions, as exact modification types weren’t ascertained.

**Fig S6**. Known archaeal tRNA modifications and their misincorporation (MI) rates compared to predicted sites of modification for *P. furiosus* and *T. kodakarensis.* A) Misincorporation (MI) frequencies of known (black points) and newly predicted (blue-orange gradient) tRNA modifications in *P. furiosus*. The x-axis lists individual modification types, and the y-axis shows their observed or predicted MI frequency. The bar plot at the top displays the total counts for each category, with color gradients corresponding to adjusted p-values. B) Equivalent MI frequency plot for *T. kodakarensis*, again showing known (black) and predicted (blue-orange) tRNA modifications, along with total counts in the bar plot above. False-positive predictions are highlighted along with their corresponding explanations. In brief, these are composed of unannotated modifications and non-random sequencing artifacts. Generally, the misincorporation frequencies of predicted modifications closely matched those of experimentally validated sites. However, several notable exceptions (“false positives”) emerged. Specifically, multiple cytosine positions exhibited significant adjusted p-values, despite lacking previous annotations. For example, position C17, a site known to be modified with either 2'-O-methylcytidine (Cm) or 2-thiocytidine (s^2^C), was not significantly annotated with Cm17 in any clade-specific reference species, resulting in these modifications at analogous positions (such as C6, C17, and C42) in *P. furiosus* and *T. kodakarensis* being called as false positives. Similarly, guanine and uridine-derived false positives are called specifically at positions known to be modified (U8, U39, G18, and G51) with 4-thiouridine (s^4^U), 2-thiouridine (s^2^U), and 2’-O-methylguanine (Gm). These signals likely represent genuine modifications that escaped homologous-prediction annotations rather than true false positives, and suggest the possible presence of some previously undetected modifications within these species. Lastly, there are occasional false positives that occur as a consequence of upstream modifications followed by short repetitive nucleotide sequences that we have termed “shadow” misincorporations. For example, G34 of Pro^GGG^ is the most commonly seen shadow across all species. Similarly, some U5 of tRNA^Gly^ and G5 of tRNA^Thr^ are also observed to present these shadows.

**Fig S7**. Coordinated modification pattern of modification in positions 10 and 26 across archaeal tRNAs. Paired misincorporation frequencies are shown for individual tRNAs predicted to be modified at position 10 and/or position 26 across all species. A) Thermococci and Thermoprotei species (*S. islandicus, S. acidocaldarius, P. furiosus, T. species* AM4, *T. kodakarensis*) exhibit co-occurrence of modifications at both positions within the same tRNA. This is especially prominent in Thermoprotei, where high-confidence predictions indicate that many tRNAs, such as tRNA^Asp^, tRNA^Glu^, and tRNA^Val^, carry both m^2^_2_G10 and m^2^_2_G26. In contrast, Thermococci species display a similar trend, but with most misincorporations occurring at position 10, suggesting that m^2^G10 is paired with m^2^_2_G26 in the same tRNA. B) In Halobacteria (*H. salinarum, H. volcanii*) and Methanococci (*M. maripaludis, M. jannaschii*), positions 10 and 26 are more often modified in mutually exclusive subsets of tRNAs, suggesting distinct substrate preferences or competitive enzyme activity. Each line represents one tRNA, connecting MI frequencies at position 10 (left) and 26 (right), with point color indicating isotype identity. Numbers above each panel reflect the number of predicted modifications at the position. Supporting data for these plots is available in Table S6.

**Fig S8**. Conserved D-stem pairing patterns associated with predicted m^2^_2_G10 and xG26 modifications across archaeal tRNAs. Each map illustrates the frequency of specific D-stem base pairings in tRNAs predicted to be modified as position 10 (panel A) or position 26 (panel B), across nine representative archaeal species. Counts represent the number of predicted modified tRNAs that contain the indicated nucleotide pairs at canonical D-stem positions: 10-25, 11-24, 12-23, and 13-22. A) tRNAs predicted to carry m^2^_2_G10 are strongly associated with G10oU25 base pair, a known identity element for Trm11-mediated modification. Additional motifs such as U13oU22 and U13oA22 are frequently observed in Euryarchaeota species (e.g., *H. volcanii, M. jannaschii*) and may contribute to substrate specificity. In contrast, some Thermoprotei species (e.g., *S. acidocaldarius*, *S. islandicus*) exhibit m^2^_2_G10 modifications in tRNA that lack these canonical features, suggesting relaxed or alternative recognition in this clade. B) xG26-modified tRNA (m^2^G, m^2^_2_G, m^2^_2_Gm) show a broader set of D-stem pairing preferences. While canonical G10oU25 pairs are common in many species, they tend to exclude position 26 modification in Euryarchaeota, consistent with prior findings in *P. furiosus*. This exclusionary relationship is not maintained in Thermoprotei, which frequently exhibit co-modification at both positions and show a preference for alternative motifs, such as C12=G23 or A12oU23. These data emphasize the strong evolutionary conservation and divergence of D-arm identity elements that guide enzyme-substrate recognition. Patterns suggest that shared structural features enable coordinated modification at positions 10 and 26 in some lineages, while other species may exhibit unique targeting.

**Fig S9**. Total tRNA read counts across three biological replicates for *H. volcanii* and *T. kodakarensis*. Two pairs of panels for each species. Top panel includes reads from each replicate, bottom panel is a summary of each, with mean and standard deviation (std).
